# An 8-Week Very Low-Calorie Ketogenic Diet (VLCKD) Alters the Landscape of Obese-Derived Small Extracellular Vesicles (sEVs), Redefining Hepatic Cell Phenotypes

**DOI:** 10.3390/nu16234189

**Published:** 2024-12-04

**Authors:** Francesco Balestra, Maria De Luca, Giorgia Panzetta, Nicoletta Depalo, Federica Rizzi, Rita Mastrogiacomo, Sergio Coletta, Grazia Serino, Emanuele Piccinno, Dolores Stabile, Pasqua Letizia Pesole, Valentina De Nunzio, Giuliano Pinto, Nicole Cerabino, Martina Di Chito, Maria Notarnicola, Endrit Shahini, Giovanni De Pergola, Maria Principia Scavo

**Affiliations:** 1 Laboratory of Molecular Medicine, National Institute of Gastroenterology IRCCS “S. de Bellis”, Via Turi 27, Castellana Grotte, 70013 Bari, Italy; francesco.balestra@irccsdebellis.it (F.B.); maria.deluca@irccsdebellis.it (M.D.L.); giorgia.panzetta@irccsdebellis.it (G.P.); grazia.serino@irccsdebellis.it (G.S.); emanuele.piccinno@irccsdebellis.it (E.P.); 2 Institute for Chemical-Physical Processes, Italian National Research Council (IPCF)-CNR SS Bari, Via Orabona 4, 70125 Bari, Italy; n.depalo@ba.ipcf.cnr.it (N.D.); f.rizzi@ba.ipcf.cnr.it (F.R.); 3 National Interuniversity Consortium of Materials Science and Technology (INSTM), Bari Research Unit, Via Orabona 4, 70126 Bari, Italy; 4 Department of Chemistry, University of Bari, Via Orabona 4, 70125 Bari, Italy; rita.mastrogiacomo@uniba.it; 5 Core Facility Biobank, National Institute of Gastroenterology “S. de Bellis”, IRCCS Research Hospital, Via Turi 27, Castellana Grotte, 70013 Bari, Italy; sergio.coletta@irccsdebellis.it (S.C.); dolores.stabile@irccsdebellis.it (D.S.); letizia.pesole@irccsdebellis.it (P.L.P.); 6 Laboratory of Nutritional Biochemistry, National Institute of Gastroenterology, “S. de Bellis”, Via Turi 27, Castellana Grotte, 70013 Bari, Italy; valentina.denunzio@irccsdebellis.it (V.D.N.); giuliano.pinto@irccsdebellis.it (G.P.); maria.notarnicola@irccsdebellis.it (M.N.); 7 Center of Nutrition for the Research and the Care of Obesity and Metabolic Diseases, National Institute of Gastroenterology IRCCS “Saverio de Bellis”, Via Turi 27, Castellana Grotte, 70013 Bari, Italy; nicole.cerabino@irccsdebellis.it (N.C.); martina.dichito@irccsdebellis.it (M.D.C.); giovanni.depergola@irccsdebellis.it (G.D.P.); 8 Gastroenterology Unit, National Institute of Gastroenterology IRCCS “S. de Bellis”, Via Turi 27, Castellana Grotte, 70013 Bari, Italy; endrit.shahini@irccsdebellis.it

**Keywords:** VLCKD, fibrosis, liver disease, small extracellular vesicles, obesity

## Abstract

**Background.** Very low-calorie ketogenic diets (VLCKD) are an effective weight-loss strategy for obese individuals, reducing risks of liver conditions such as non-alcoholic steatohepatitis and fibrosis. Small extracellular vesicles (sEVs) are implicated in liver fibrosis by influencing hepatic cell phenotypes and contributing to liver damage. This study investigates sEVs derived from serum of 60 obese adults categorized into low fibrosis risk (LR) and intermediate/high fibrosis risk (IHR) groups based on FibroScan elastography (FIB E scores, limit value 8 kPa) and all participants underwent an 8-week VLCKD intervention. **Methods.** The study examines the impact of these sEVs on fibrosis markers, inflammation, and autophagy in a hepatocyte cell line (HEPA-RG) using bioinformatics, RNA sequencing, lipidomics, RT-PCR, and Western blotting before (T0) and after (T1) VLCKD. **Results.** sEVs from LR patients post-VLCKD reduced fibrosis related gene expression (e.g., ACTA2) and enhanced proteins associated with regeneration and inflammation (e.g., HDAC6). Conversely, sEVs from IHR patients increased fibrosis and inflammation related gene expression (PIK3CB, AKT1, ACTA2) in hepatocytes, raising concerns about VLCKD suitability for IHR patients. IHR sEVs also decreased expression of HDAC10, HDAC6, HDAC3, MMP19, and MMP2, while increasing modulation of p-AKT, α-SMA, and VIM. **Conclusion.** These findings underscore the critical role of sEVs in regulating inflammation, remodeling, and hepatic stress responses, particularly in IHR patients, and suggest sEVs could complement instrumental evaluations like FibroScan in fibrosis assessment.

## 1. Introduction

In individuals who suffer from obesity, the location of fat deposits is more critical than total body weight in terms of metabolic complications that contribute to comorbid conditions [1]. Specifically, visceral adipose tissue build-up is associated with increased peripheral insulin resistance and often leads to a condition known as lipo-inflammation, a chronic low-grade inflammatory state [2]. Similarly, the accumulation of fat in the liver is associated with peripheral insulin resistance and localized inflammation, leading to persistent damage to liver cells. This condition is known as Metabolic Dysfunction-Associated Steatotic Liver Disease (MASLD) [3]. The interaction between different fat stores and the consequent metabolic disruptions can ultimately lead to organ damage and raise the risk of cardiovascular diseases in individuals with obesity. In some cases, chronic inflammation in the liver can progress to more severe conditions such as non-alcoholic steatohepatitis and fibrosis, potentially culminating in liver failure and a higher risk of hepatocellular carcinoma [4]. Transient elastography, or FibroScan, is a widely used ultrasound technique for assessing hepatic steatosis [5]. It provides the FibroScan elastography (FIB E) value, with levels below 8 kPa indicating minimal fibrosis and low risk of liver degeneration, while values above 8 kPa suggest a high risk of chronic liver disease [6]. Among commonly used evaluation techniques, the concept of magnetic resonance imaging (MRI) as a “Virtual Liver Biopsy” has emerged. This approach, allow the acquisition of data from MR elastography, liver fat quantification, and liver iron quantification. Furthermore, these techniques are typically integrated with a standard gadolinium-enhanced abdominal MRI examination, offering the most comprehensive liver imaging currently available [7,8]. MASLD prevention focuses on lifestyle changes, including a healthy diet and increased physical activity. Obesity-associated “low-grade” inflammation, driven by decreased anti-inflammatory cytokines and elevated pro-inflammatory molecules, contributes to MASLD and may lead to liver damage or cirrhosis [9,10]. Early-stage MASLD is reversible with lifestyle modifications and addressing causative factors [11]. Weight loss remains the most effective treatment for inflammation. High-quality diets, such as the Dietary Approaches to Stop Hypertension (DASH) diet and the Mediterranean diet, have demonstrated efficacy in reducing liver steatosis and systemic inflammation. Currently, the primary treatment for these patients involves weight loss and lifestyle modifications, including greater physical activity and calorie-restricted diets. However, traditional low-calorie diets and exercise often fail to bring about the significant weight loss (> 5%) required to reduce liver fat [12]. The very low-calorie ketogenic diet (VLCKD) has been proposed as an effective weight loss strategy for obese patients and individuals to decrease visceral adipose tissue and insulin resistance and damage to organs [13]. The effects on the risk of progression to more severe liver diseases, such as fibrosis or Metabolic dysfunction Associated SteatoHepatitis (MASH), remain less clear. Non-invasive tools such as FibroScan represent the standard for assessing fibrosis risk but do not provide insights into the underlying molecular mechanisms, and if the benefits of VLCKDs for the treatment of obesity are well known, a detailed understanding of how these interventions influence molecular mechanisms in patients with varying levels of fibrosis risk is lacking. Recent studies have indicated that extracellular vesicles (EVs) contribute to the progression of liver fibrosis by promoting hepatic cell degeneration and activating hepatic stellate cells [14,15]. Extracellular vesicles (EVs) are membrane-bound structures secreted by cells into the extracellular space. They are essential for intercellular communication and have recently been categorized into distinct groups based on their size and origin, including a subset known as sEVs. The biogenesis of sEVs primarily involves two main pathways: the endosomal pathway and direct budding of the plasma membrane. The formation of sEVs starts with the inward budding of the plasma membrane, leading to the creation of early endosomes. These progress to late endosomes, which then undergo further inward budding to produce multivesicular bodies containing intraluminal vesicles. When multivesicular bodies merge with the plasma membrane, intraluminal vesicles with diameters smaller than 200 nm [16] are released into the extracellular environment as sEVs [17]. Another mechanism involves direct outward budding and fission of the plasma membrane, resulting in the release of ectosomes. This process is regulated by changes in calcium levels and cytoskeletal dynamics, influencing membrane curvature and vesicle formation [18,19].

Actually, it is unclear whether VLCKDs have differential effects on sEVs based on the initial fibrosis risk, and how these effects might influence liver remodeling. The potential use of molecular biomarkers, such as those carried by sEVs, could enhance the diagnosis and monitoring of liver diseases.

This pilot study focuses on the role of sEVs derived from the serum of 60 adult patients enrolled at the Center of Nutrition for Obesity and Metabolic Diseases of our institute. At the time of enrollment, patients neither consumed alcohol nor exhibited contraindications to a very low-calorie ketogenic diet (VLCKD), such as specific medical conditions including type 1 diabetes, cardiovascular and respiratory diseases, gastrointestinal disorders, kidney diseases, or pregnancy and lactation. These participants were classified as Low Risk (LR) and Intermediate High Risk (IHR), based on their FIB E scores, and were enrolled in an 8-week weight loss program utilizing a VLCKD. This study aims to integrate the insights provided by FibroScan with a comprehensive molecular analysis of sEVs to advance the understanding and management of liver diseases in obese patients. It focuses on an in-depth investigation of the influence of sEVs on cellular communication during the regeneration and degeneration processes of human hepatic cells (HEPA-RG). By highlighting the role of sEVs as mediators and biomarkers, the research seeks to support the development of targeted therapeutic interventions and the creation of complementary diagnostic tools. This was analyzed by assessing cellular stress fibers and their associated molecules, using experimental procedures designed to detect variations in the transcriptomic, lipidomic, genetic, and proteomic profiles of hepatocytes treated with sEVs derived from patients before and after VLCKD intervention. This study goal was to investigate the role of sEVs derived from serum of 60 obese adults without any comorbid conditions.

## 2. Materials and Methods

### 2.1. Patients

The study, spanning eight weeks, was carried out at the Center of Nutrition for Obesity and Metabolic Diseases, part of the National Institute of Gastroenterology “Saverio De Bellis” Research Hospital in Castellana Grotte, Bari, Italy. A total of 60 patients were enrolled in the study. Participants were excluded if they consumed excessive alcohol (more than 20 g/day for women and 30 g/day for men) or presented contraindications for a very low-calorie ketogenic diet (VLCKD), such as specific medical conditions, including type 1 diabetes, severe cardiovascular or respiratory diseases, gastrointestinal disorders, chronic kidney disease, psychiatric disorders, or if they were pregnant or lactating. Eligible participants were aged between 18 and 64 years, had a Body Mass Index (BMI) ≥ 30 kg/m², and were not undergoing any integrative supplemental or pharmaceutical treatment at the time of the study. Exclusion criteria extended to contraindications for VLCKD, severe medical or psychiatric conditions, eating disorders, substance abuse, active infections, and rare disorders. Additionally, daily alcohol consumption and smoking habits were assessed through direct questioning, adhering to established American and European guidelines on alcohol intake. The local Medical Ethics Committee approved the study (approval number 179/C.E. de Bellis, issued on May 13, 2022), that was conducted in compliance with the ethical standards of the 1964 Declaration of Helsinki. All participants provided written informed consent. The study was registered on ClinicalTrials.gov (identifier: NCT05477212). Patient recruitment took place from July 2022 to December 2023, with evaluations conducted at baseline (T0) and after eight weeks (T1) of VLCKD treatment. The VLCKD protocol adhered to the guidelines outlined by Rinaldi et al. [20]. Participants adhered to a daily intake of 650–800 kcal, consisting of 20 g of fat and less than 30 g of carbohydrates derived primarily from vegetables. Olive oil served as the main fat source, and participants were instructed to consume two liters of water daily. Micronutrient supplementation was provided to ensure nutritional adequacy. Adherence to the dietary protocol was closely monitored through the maintenance of dietary diaries. At T0 and T1, blood samples were collected to analyze biochemical markers, including Aspartate Aminotransferase (AST), Alanine Aminotransferase (ALT), Gamma-Glutamyl Transferase (γ-GT), fasting blood glucose, triglycerides, total cholesterol, Low-Density Lipoproteins (LDL), and High-Density Lipoproteins (HDL). BMI was measured using a calibrated scale and stadiometer, and body composition was assessed via Bioelectrical Impedance Analysis. Liver fibrosis and steatosis were evaluated through transient elastography (FibroScan^®^ Expert 630, Echosen Italia SRL, Verona, Italy), performed after a minimum fasting period of six hours. Measurements were conducted on the right liver lobe, capturing liver stiffness (kPa) and Controlled Attenuation Parameter (dB/m) values. Reliable results were defined as having an interquartile range of less than 30% of the median and at least ten valid measurements. This comprehensive study protocol enabled a detailed evaluation of the effects of an eight-week VLCKD on liver health and metabolic parameters in obese individuals. By incorporating clinical assessments, biochemical analyses, and advanced imaging techniques, the study provides valuable insights into the metabolic and hepatic benefits and potential limitations of VLCKD in the management of obesity-related conditions.

### 2.2. sEVs Extraction and Characterization

sEVs were isolated from patients’ plasma following a modified protocol adapted from previous methods [21]. Plasma was initially centrifuged at 1800× *g* for 10 min at 4 °C, and the supernatant was transferred to a new tube and centrifuged at 3800× *g* for 15 min at 4 °C. The supernatant obtained was transferred again and centrifuged at 12500× *g* for 15 min at 4 °C. After, the supernatant underwent ultracentrifugation using a BECKMAN (Brea, CA, USA) L-60 Ultracentrifuge at 75,000× *g* for 1 h at 4 °C. The final supernatant was transferred to a fresh ultracentrifuge tube for a second ultracentrifugation at 100,000× *g* for 2 h. The resulting pellet, containing the purified sEVs, was resuspended in 200 μL of ultrapure water. Freshly isolated sEVs were analyzed using Dynamic Light Scattering (DLS), ζ-potential measurements, and Transmission Electron Microscopy (TEM). Remaining samples were stored at −80 °C for subsequent protein analysis. TEM analysis was conducted with a Jeol Jem-1011 electron transmission microscope (JEOL USA, Inc., Peabody, MA, USA) operating at 100 kV, and images were captured using an Olympus Quemesa Camera (11 MP) (Olympus, Shinjuku-ku, Tokyo, Japan). Hydrodynamic diameter, size distribution, and colloidal stability of the sEVs were evaluated using a Zetasizer Nano ZS (Malvern Instruments Ltd., Worcestershire, UK) with DTS 5.00 software. Results are presented as mean ± standard deviation from three independent replicates.

### 2.3. Cell Culture

The HEPA-RG human hepatoma cell line (Thermo Fisher Scientific, Waltham, MA, USA) was cultured using a hepatocyte bullet kit medium composed of: Transferrin 0.5 mL, Ascorbic Acid Solution 0.5 mL, HEGF 0.5 mL, Recombinant Human Insulin 0.5 mL, Hydrocortisone 0.5 mL, BSA/Fatty Acid Free 10 mL, GA-1000 0.5 mL from Lonza Biowhittaker (Oslo, Norway) supplemented with 10% exosome-depleted FBS from Lonza Biowhittaker (Oslo, Norway) and 1% Antibiotic-Antimycotic (penicillin 10,000 U/mL, streptomycin 10,000 U/mL) from the same supplier. For treatments with sEVs, the concentration used was 20 μg/μL of sEVs proteins derived from patients with LR or IHR for chronic liver disease for 8, 24, 48, or 96 h, depending on the specific experimental requirements for the transcriptomic, lipidomic or protein functionality study. For each experiment, an appropriate number of cells were used and stimulated, particularly, for lipidomic and protein studies, cells were seeded in 6-well plates and stimulated when they reached semi-confluence (1 × 10⁶ cells), with stimulation occurring every 24 h. For experiments involving RNA, cells were seeded in 12-well plates and similarly stimulated at semi-confluence (0.5 × 10⁶ cells) every 24 h. For cell viability experiments, HEPA-RG cells were seeded in 96-well plates at a density of 2 × 10³ cell per well, and stimulated after 24 h and every 24 h with sEVs.

### 2.4. Study of Lipidomic Profile of HEPA-RG Treated with Patients’ sEVs Before and After VLCKD Treatment

The fatty acid profile in membranes of HEPA-RG cell line treated with sEVs from all subjects was evaluated using the Moilanen method [22], modified from Folch’s method [23]. After 72 h of treatment, total fatty acids were extracted from the cells. Fatty acids were hydrolysed from cell membrane phospholipids by adding 450 µL of an acidified salt solution (H_2_SO_4_ 2 × 10^−4^ M, NaCl 0.1%). Then, 2.25 mL of a chloroform (2:1, *v/v*) mixture (Sigma-Aldrich, Milan, Italy) was added, mixed well, and centrifuged at 1000× *g* for 20 min. The lower layer containing fatty acids was collected, dried with a centrifugal evaporator (Thermo Fisher Scientific, Milan, Italy), and converted to fatty acid methyl esters (FAME) by adding 250 µL of toluene and 750 µL of BF3·MeOH 14% (Sigma-Aldrich, Milan, Italy), followed by incubation at 80 °C for 2 h. Each sample was then mixed with 1.25 mL of 5% aqueous sodium chloride solution and 250 µL of toluene, and centrifuged at 470× *g* for 10 min. The upper layer containing FAMEs was collected for gas chromatography (GC) analysis. Fatty acid quantification was performed using a Thermo Fisher Scientific GC with an autosampler, split/split-less injector, FID detector, and hydrogen gas generator (Thermo Fisher Scientific, Milan, Italy). Separation of FAMEs was achieved using a BPX 70 capillary column (SGE Analytical Science, 60 m × 0.25 mm ID, 0.25 µm, SGE Europe Ltd., Milton Keynes, UK). Hydrogen was used as the carrier gas at a constant flow of 3.0 mL/min. The injector and FID detector temperatures were maintained at 250 °C. The oven temperature program was initially 40 °C, then ramped up to 170 °C at 10 °C/min, held for 5 min, increased to 200 °C at 4 °C/min, held for 5 min, and finally increased to 240 °C at 10 °C/min, held for 5 min. FAMEs were quantified using a standard mixture (Supelco 37 Component FAME Mix, Sigma-Aldrich, Milan, Italy).

### 2.5. RNA Extraction

The total RNA from HEPA-RG treated with sEVs from all subjects for 8 h, was extracted with the RNeasy^®^ Mini kit (QIAGEN, Hilden, Germany). Briefly, following the vendor’s protocol, 350 µL of RLT buffer (included in the kit) was added to the cells collected from each well of a 12-well plate. The solution was vortexed, and 70% ethanol (1:1 *v/v*) was added. After gentle pipetting, 700 µL of each sample was transferred to a RNeasy Mini Spin Column and centrifuged for 15 s at 8000× *g*. The flow-through was discarded, and 350 µL of RW1 buffer (Qiagen kit) was added to the column, followed by centrifugation for 15 s at 8000× *g*. After removing the flow-through, 10 µL of DNase I stock solution and 70 µL of RDD buffer were mixed by inverting the tube and briefly centrifuged. Subsequently, 80 µL of DNase I solution was added to each column and incubated at room temperature for 15 min. Next, 350 µL of RW1 buffer was added, mixed, and centrifuged for 15 s at 8000× *g*. After discarding the flow-through, three washes were performed with 500 µL of RPE buffer, centrifuging each time for 15 s at 8000× *g*. The collection tubes were replaced with clean ones, and the columns were centrifuged at 15,000× *g* for 1 min. Columns were then transferred to new 1.5 mL tubes, and 40 µL of RNase-free H_2_O was added to elute the RNA. The columns were centrifuged for 1 min at 8000× *g* to collect the RNA eluate. The RNA concentration was determined using the Qubit™ RNA HS Assay Kit (Thermo Fisher Scientific, Waltham, MA, USA) on a Qubit Fluorometer (Thermo Fisher Scientific, Waltham, MA, USA). RNA quality was assessed with the High Sensitivity RNA ScreenTape (Agilent Technologies, Palo Alto, CA, USA) and analyzed using the Agilent 4200 TapeStation system (Agilent Technologies, Palo Alto, CA, USA).

### 2.6. RNA-Sequencing on HEPA-RG Treated with Patients’ sEVs and Bioinformatics Analysis

cDNA was synthesized using the Ion Torrent™ NGS Reverse Transcription Kit (Thermo Fisher Scientific, Waltham, MA, USA) following the manufacturer’s instructions. Target region amplification was carried out with the Ion AmpliSeq Transcriptome Human Gene Expression core panel (Thermo Fisher Scientific, Waltham, MA, USA) using the Ion Chef System. The resulting libraries were quantified using the Ion Library TaqMan Quantitation Kit (Thermo Fisher Scientific, Waltham, MA, USA). Libraries were prepared with the Ion Chef, and sequencing was performed on a 540 chip using the Ion GeneStudio S5 Prime system (Thermo Fisher Scientific, Waltham, MA, USA). The sequencing data are accessible under accession number GSE279581 in the Gene Expression Omnibus database [http://www.ncbi.nlm.nih.gov/geo/ URL (accessed on 16 October 2024)]. Raw read counts were generated using the ampliSeqRNA plugin within the Ion Torrent Suite Server v5.16.1 software (Thermo Fisher Scientific, Waltham, MA, USA). Subsequent analyses were conducted using Transcriptome Analysis Console 4.0 software (Thermo Fisher Scientific, Waltham, MA, USA). Differentially expressed genes (DEGs) were identified with a fold-change threshold of 1.5 and a *p*-value of ≤0.05. Hierarchical clustering was performed using Alt Analyze 2.1.3 [24].

Interactions among significant identified genes were predicted with the Search Tool for the Retrieval of Interacting Genes/Proteins (STRING) database version 11.0.

### 2.7. cDNA Synthesis, and PCR Analysis

Gene expression analysis was performed on the treated HEPA-RG cell line to evaluate several genes: PIK3CB, AKT1, HDAC 10, HDAC3, HDAC6, MMP19, MMP9, MMP2, VIM, and ACTA2 were analysed using the specified Bio-Rad protocol. Briefly, total RNA was extracted with the RNeasy Mini Kit (50) (Qiagen); 2 µg of RNA were used for reverse transcription using the High-Capacity cDNA Reverse Transcription kit (200 reaction) (Thermo Fisher). Quantitative real-time PCR was performed on 10 ng/µL c-DNA with iTaq™ Universal SYBR^®^ Green Supermix (Bio-Rad) and the CFX96 Touch™ qPCR System (Bio Rad). Thermal cycling conditions were 95 °C for 2 min, 39 cycles at 95 °C for 5 s and 60 °C for 30 s; 95 °C for 5 s, from 65 °C to 95 °C for 5 s with 0.5 °C increments. Gene analyses were performed with the pre-validated Bio-Rad primers PIK3CB qHsaCED0056640, AKT1 qHsaCID0011338, HDAC3 qHsaCED0046833, HDAC6 qHsaCID0015009, HDAC10 qHsaCED0037957, MMP2 qHsaCID0015623, MMP9 qHsaCID0011597, MMP19 qHsaCED0043982, VIM qHsaCID0012604, ACTA2 qHsaCID0013300, GAPDH qHsaCED0038674 (Bio-Rad). The relative gene expression was calculated by comparing the expression levels of the genes of interest to the housekeeping gene GAPDH using the 2^(-ΔΔCt) method.

### 2.8. Western Blotting Analysis

Western blotting was performed to analyze protein expression in HEPA-RG cells after 48-hour exposure to sEVs. Target proteins included protein kinase-B (AKT), phospho-protein kinase-B (*p*-AKT), phosphoinositide 3-kinase-B (PI3KB), protein histone deacetylase-3 (HDAC3), histone deacetylase-6 (HDAC6), matrix metalloproteinase 2 (MMP2), matrix metalloproteinase 9 (MMP9), vimentin (VIM), and alpha-smooth muscle actin (α-SMA).

Proteins were extracted using RIPA buffer with protease and phosphatase inhibitors (Thermo Scientific, Rockford, IL, USA), and concentrations measured via Bradford assay (Bio-Rad, Milan, Italy). Aliquots of 30 µg of total protein extracts were separated on 4–15% polyacrylamide gels and transferred onto polyvinylidene fluoride (PVDF) membranes (Bio-Rad Laboratories, Milan, Italy).

The membranes were incubated with primary antibodies against PIK3CB, AKT, *p*-AKT, MMP2, MMP9, VIM, HDAC3, and HDAC6 (1:500 dilution, Cell Signaling Technology, Beverly, MA, USA), anti-α-SMA (1:400, Thermo Fisher), and anti-glyceraldehyde-3-phosphate dehydrogenase (GAPDH) (1:1000, Santa Cruz, Santa Cruz, CA, USA). After washing, secondary antibodies were applied, and proteins detected using chemiluminescence (ECL, Thermo Scientific, Rockford, IL, USA). Signals were captured with a ChemiDoc Molecular Imager (Bio-Rad, Milan, Italy) and normalized to GAPDH, with analysis conducted via Image Lab 5.2.1 software (Bio-Rad, version 4.6).

### 2.9. Cell Viability of HEPA-RG After Treatment with sEVs

The hepatic cell line HEPA-RG was seeded into 96-well plates at a density of 2 × 10³ cells per well. After 24 h, the cells were treated with sEVs derived from the sera of LR and IHR patients before and after 8 weeks of VLCKD administration, respectively T0 and T1, and incubated at 37 °C for 96 h, with patient-derived sEVs stimulation every 24 h. Untreated cells served as controls. Following sEVs treatment (96 h), the cell line was incubated with 20 μL of MTS tetrazolium reagent ([3-(4,5-dimethylthiazol-2-yl)-5-(3-carboxymethoxyphenyl)-2-(4-sulfophenyl)-2H-tetrazolium], CellTiter 96^®^ AQueous One Solution Cell Proliferation Assay, Promega, Madison, WI, USA) in 100 μL of medium for a total volume of 120 μL at 37 °C for 3 h with 5% CO_2_. The quantity of formazan product, measured by absorbance at 490 nm, was directly proportional to the number of living cells in culture and the was measured using a PerkinElmer Victor Plate Reader (Lier, Belgium).

### 2.10. Statistical Analysis

The Shapiro-Wilk test was used to evaluate the normality of the data distribution and therefore parametric tests were chosen. In addition, the proportion test was used to evaluate the homogeneity between independent groups about gender parameter. Statistical analysis was performed using one-way Analysis of Variance (ANOVA) to assess the differences among the groups. For all analyses, control subjects at baseline were used as the reference group. Statistical significance thresholds were set at * *p* < 0.05, ** *p* < 0.001 and *** *p* < 0.0001. Post-Hoc analysis was carried out using GraphPad Prism, version 5.0 (GraphPad Software, San Diego, CA, USA); in particular, Bonferroni test or T-test were used for adjustments in multiple comparisons.

## 3. Results

### 3.1. Patients

**Figure 1:**
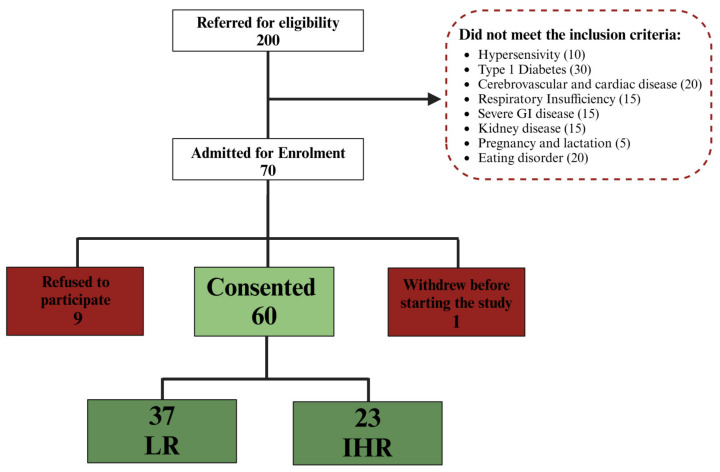
Flow diagram of patients enrolment. Of 200 potential participants, 70 met the inclusion criteria. Of these, 9 declined to participate, and another 1 withdrew at the beginning after the start of the treatment. In the end, 60 patients, 37 patients were classified as LR and 23 as IHR, were enrolled and successfully completed the treatment.

Patient classification was performed based on their responsiveness to the VLCKD, primarily utilizing fibrosis assessment through the Fibro-Scan value measured in kilopascals (kPa), which facilitated the determination of FIB E scores. In particular, our patients were 43.2% and 47.8% for LR and IHR respectively and no differences were observed between the independent groups. Furthermore the patients with a FIB E < 8 were defined as Low Risk patients (LR), while patients with FIB E ≥ 8 were defined as Intermediate-High Risk (IHR) for chronic liver disease. This Fibro-Scan parameter was measured before (T0) and after 8 weeks of treatment with VLCKD (T1). Firstly, the physician reviewed each patient’s dietary diary to verify their adherence to the diet. As shown in the scheme reported in Figure 1, 60 patients were enrolled after the evaluation of exclusion criteria, and 37 were classified as LR due to a FIB E value < 8, while 23 patients were classified as IHR, with FIB E ≥ 8.

The values presented in Table 1 indicate a statistically significant reduction in both the LR and IHR groups in terms of weight, BMI, fasting blood glucose, LDL, and HDL levels (*p* < 0.0001 to *p* < 0.005). Total cholesterol levels were significantly lower only in the LR group, while FIB E (kPa) and triglyceride levels showed a significant reduction exclusively in the IHR group, although a notable decrease was also observed in the LR group. The analysis considers the mean values for all patients.

### 3.2. sEVs Characterization

**Figure 2:**
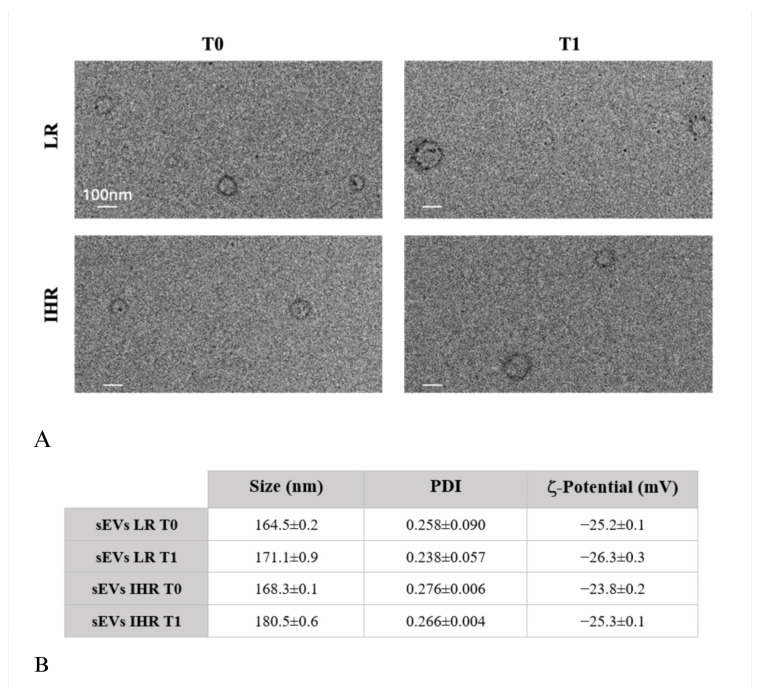
sEVs were isolated from plasma of LR and IHR patients, prior to the dietary intervention (T0) and following 8 weeks of VLCKD (T1): TEM micrographs, scale bar 100 nm (**A**) and table reporting the intensity-weighted average hydrodynamic diameter and corresponding polydispersity index (PDI) obtained by DLS analysis, along with ζ-potential values (**B**).

DLS, TEM, and ζ-potential measurements were performed to characterize sEVs isolated from the plasma of LR and IHR patients both prior to the dietary intervention (T0) and after 8 weeks of a VLCKD regimen (T1). These analyses aimed to assess the size, size distribution, morphology, and surface charge of the sEVs. As depicted in Figure 2A, TEM micrographs confirmed the presence of bilayer membrane-bound nanostructures with a spherical morphology, characteristic of sEVs, and sizes consistent with the results obtained from DLS analysis (Figure 2B). ζ-potential measurements further indicated a negative surface charge on the sEVs, which aligns with the phospholipid composition of their membrane.

### 3.3. Fatty Acid Assessment of HEPA-RG After Treatment with sEVs

**Figure 3:**
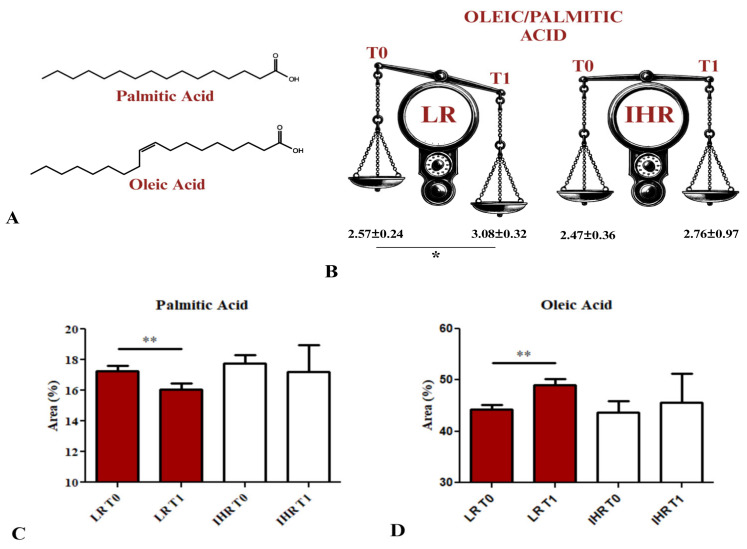
Lipidomic evaluation. Chemical structures of palmitic and oleic acid (**A**). Comparison of oleic/palmitic acid ratio obtained by GC-FID chromatography of fatty acid extracted from cell membranes of HEPA-RG treated with sEVs from patients with LR and patients with IHR prior to the dietary intervention (T0) and following 8 weeks of VLCKD (T1) (**B**) (* *p* < 0.05). Area % of palmitic acid and oleic acid determined by GC-FID chromatography in cell membranes of HEPA-RG treated with sEVs from patients with LR and patients with IHR (**C**,**D**) (** *p* < 0.001).

The fatty acid composition of HEPA-RG cell membranes was analysed before (T0) and after (T1) treatment with sEVs derived from LR and IHR patients. Table S1 presents the percentage concentrations of fatty acid in cell samples from LR-derived sEVs, showing a decline in all fatty acid, in particular a significant decrease in palmitic acid (*p* = 0.0086), whereas oleic acid was the only fatty acid to increase significantly between T0 and T1 (*p* = 0.0096), as illustrated in Figure 3. In contrast, no significant changes were observed in the cell membranes after IHR-derived sEVs treatments. Furthermore, the ratio between the chromatographic peak intensities of palmitic acid and oleic acid significantly increased in LR patients, from 2.57 ± 0.24 at T0 to 3.08 ± 0.32 at T1 (*p* < 0.05), as shown in Figure 3A,B. Additionally, a significant reduction in the percentage area of palmitic acid in the HEPA-RG cell membranes treated with LR-derived sEVs (*p* < 0.005) was documented, shown in Figure 3C. Conversely, an increase in oleic acid was observed after both LR (*p* < 0.005) and IHR sEVs treatments, as reported in Figure 3D.

### 3.4. Transcrimptomic Analysis of HEPA-RG Treated with sEVs

**Figure 4:**
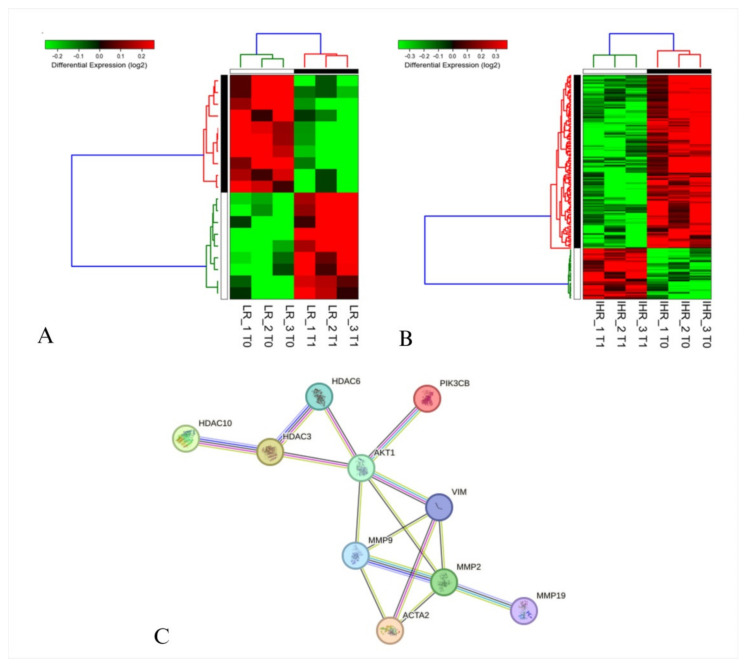
Gene expression analysis of HEPA-RG after stimulation with patient-derived sEVs before (T0) and after (T1) treatment for 8 weeks of VLCKD, using samples from both LR (**A**) and IHR (**B**) patients. Each row represents a gene, while each column corresponds to a sample. Relative expression levels are depicted using a color-coded scale, with red indicating high expression and green indicating low expression, as shown in the scale provided at the top. An interaction network (**C**) was constructed using the Search Tool for the Retrieval of Interacting Genes/Proteins (STRING). In this network, nodes represent proteins, and edges indicate predicted functional associations based on seven distinct sources of evidence: fusion events, neighborhood relationships, co-occurrence, experimental data, text mining, database information, and co-expression patterns. The color scheme is as follows: light blue indicates curated databases; light pink denotes experimentally determined interactions; gray represents co-expression; yellow signifies gene neighborhood; orange indicates gene fusions; blue denotes gene co-occurrence; green represents text mining; and purple indicates protein homology.

Transcriptomic analysis was performed to investigate the molecular changes of HEPA-RG after stimulation with patient-derived sEVs before (T0) and after (T1) treatment with a VLCKD, using samples from both IHR and LR patients. We found that HEPA-RG exhibited significant molecular alterations only when stimulated with sEVs of IHR patients, after the VLCKD treatment. In particular, the treatment of sEVs from LR patients highlighted the finding that only 19 genes were differentially expressed at T1 compared to T0 (Table S2, Figure 4A). Moreover, the stimulation with sEVs of IHR patients at T1 compared to the same patients at T0 elicited a list of 164 differentially expressed genes (Table S3). Unsupervised hierarchical clustering analysis clearly demonstrated the differences between the T0 and T1 samples (Figure 4B).

The interactions between several proteins encoded by the genes studied in the transcriptomic analysis of HEPA-RG cells treated with sEVs derived from IHR patients were examined using the STRING database. The analysis indicated that the activation of PIK3CB plays a role in modulating AKT and HDAC proteins, including HDAC3, HDAC6, and HDAC10 (evaluated in transcriptomics analysis), which are involved in hepatocyte degeneration. Meanwhile, PIK3CB is linked to MMP2, MMP9, MMP19 (evaluated in transcriptomic analysis), and VIM and α-SMA, proteins known to play roles in extracellular matrix modulation and cellular stress responses (Figure 4C).

### 3.5. Molecular Characterization of Gene Expression Involved in Hepatocyte Degeneration in Liver Fibrosis

The gene expression levels of PIK3CB, AKT1, HDAC10, HDAC3, HDAC6, MMP19, MMP2, MMP9, ACTA2 and VIM were evaluated in HEPA-RG cells using real-time PCR (RT-PCR) after 8 h of treatment with the two types of sEVs.

**Figure 5:**
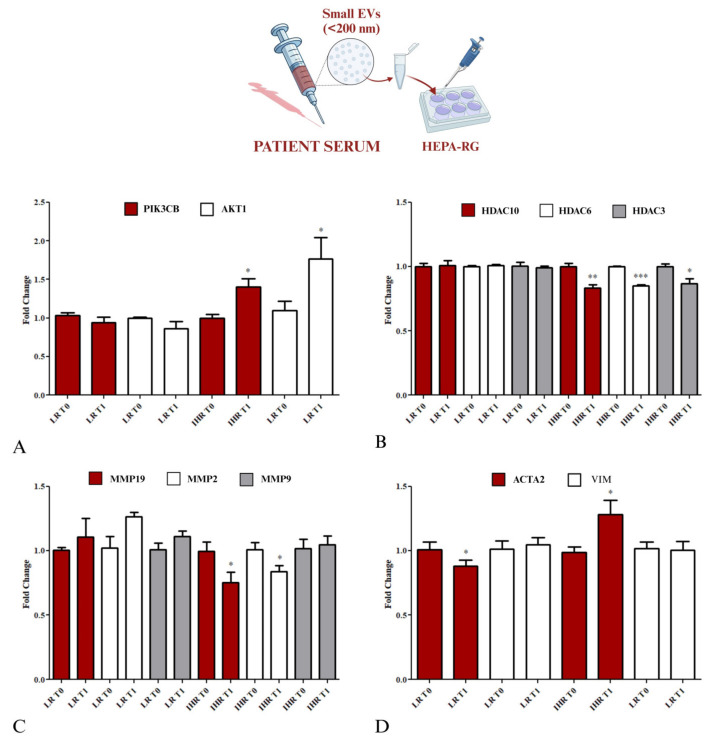
Gene expression of PIK3CB, AKT1, HDAC10, HDAC6, HDAC3, MMP19, MMP2, MMP9, ACTA2, and VIM in HEPA–RG cells treated with serum-derived sEVs from obese patients after VLCKD for 8 weeks. Patients with LR and patients with IHR at T0 (before the VLCKD) and after 8 weeks of VLCKD (T1). For the HEPA-RG expression, the fold change values for PIK3CB and AKT1 are reported in (**A**). The fold change values for HDAC 10, HDAC 6, and HDAC 3 are reported in (**B**). The fold change values for MMP19, MMP9, and MMP3 are reported in (**C**). The fold change values for VIM and ACTA2 are reported in (**D**). (* *p* < 0.05, ** *p* < 0.001, and *** *p* < 0.0001 for T1 vs. T0).

A significant increase of PIK3CB and AKT1 expression, as well as ACTA2 expression, was revealed when the cells were treated with sEVs derived from IHR patients after VLCKD (*p* < 0.05) compared to T0 (Figure 5A), while a strong reduction in the mRNA expression levels of HDAC10 (*p* < 0.0001), HDAC6 (*p* <0.001), and HDAC3 (*p* <0.05) was also observed in cells treated with the same conditions (Figure 5B).

RT-PCR analysis revealed a significant reduction in MMP19 and MMP2 expression when the cells were treated with sEVs, from IHR patients, at T1 compared to T0 (Figure 5C) (*p* < 0.05).

By contrast, no differences in mRNA expression were noted in cells treated with sEVs derived from LR patients at either time point. Additionally, the cells treated with sEVs derived from LR patients did not show modifications in gene expression of all metalloproteases analysed.

Moreover, cells treated with sEVs derived from LR patients exhibited a significant decrease in ACTA2 expression (*p* <0.05), without any notable change in VIM expression, whereas cells treated with sEVs from IHR patients showed a significant increase in ACTA2 expression after undergoing VLCKD (Figure 5D), while no differences were observed in VIM expression in these cells.

### 3.6. Expression of Proteins Involved in Hepatocyte Degeneration and in Liver Fibrosis in Cells Treated with sEVs

**Figure 6:**
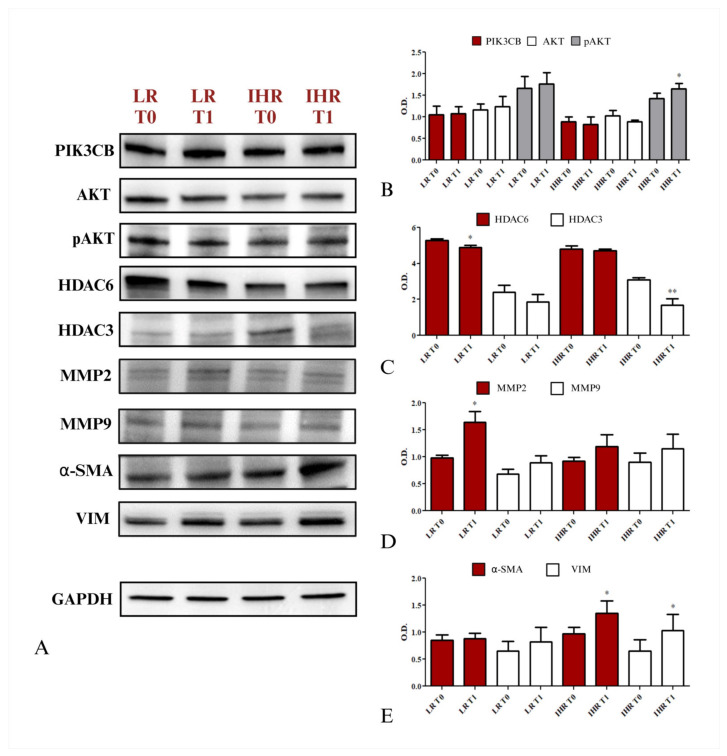
Evaluation of proteins regulating hepatocyte degeneration and liver fibrosis was conducted in HEPA-RG cell lines treated with sEVs isolated from patients with LR and patients with IHR, both before (T0) and after (T1) 8 weeks of VLCKD. Representative Western blots of various proteins (PIK3CB, AKT, pAKT, HDAC3, HDAC6, α-SMA, and VIM) and the housekeeping protein GAPDH (**A**). A semi-quantitative evaluation of protein expression levels was performed using video-densitometry analysis of PIK3CB, AKT, and pAKT1 (**B**), HDAC6 and HDAC3 (**C**), MMP2 and MMP9 (**D**), and α-SMA and VIM (**E**) bands on the Western blots. The GAPDH protein band was used to normalize the protein bands for each subject. (* *p* < 0.05 and ** *p* < 0.001 for T1 vs. T0).

The expression levels of various proteins, specifically PIK3CB, AKT, pAKT, HDAC3, HDAC6, MMP9, MMP2, VIM, and α-SMA, which are implicated in hepatocyte degeneration as well as the initiation and progression of liver fibrosis, were analyzed using Western blotting. Figure 6A reports a representative Western blot derived from HEPA-RG cells treated with sEVs from LR and IHR patients at T0 and T1. No significant alterations in protein expression levels were observed for PIK3CB and AKT. However, a significant increase of expression of pAKT was noted in cells treated with sEVs derived from IHR at T1 (*p* < 0.05) (Figure 6B).

The expression of HDAC6 was significantly reduced in HEPA-RG cells treated with LR at T1 (*p* <0.05) compared to T0, while the same treatment did not alter the expression of HDAC3. In contrast, the expression of HDAC6 did not change significantly in cells treated with IHR, whereas HDAC3 showed a strong reduction when the cells were treated with IHR sEVs at T1 compared to T0 (Figure 6C).

The analysis of MMP2 and MMP9 revealed a significant increase of MMP2 expression only in cells treated with sEVs extracted from LR patients following the VLCKD, while expression levels remained almost constant in the other cell groups. Similarly, the expression of MMP9 did not undergo any significant changes in any of the cell groups treated with sEVs derived from either LR or IHR patients (Figure 6D).

Finally, a significant increase in the expression of α-SMA and VIM was observed in HEPA-RG cells treated with sEVs from IHR patients at T1, while no such effect was noted in cells treated with sEVs from LR patients (Figure 6E)

### 3.7. Effect of sEVs Isolated from LR and IHR Patients on HEPA-RG Proliferation

**Figure 7:**
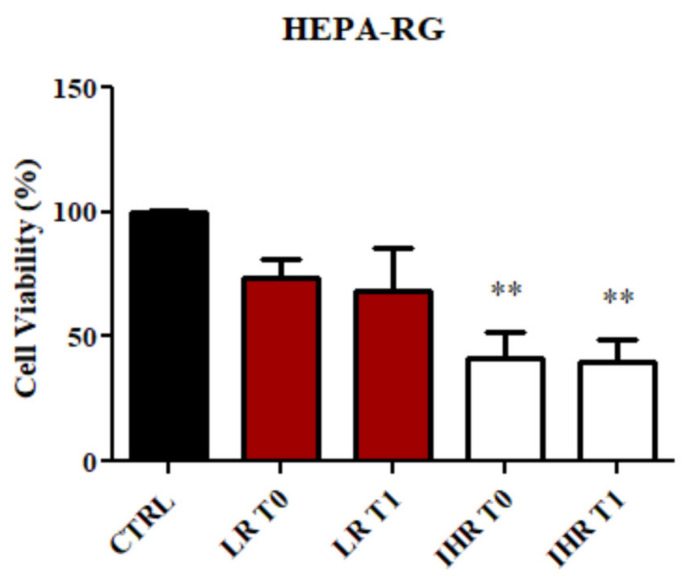
HEPA-RG cells line viability evaluation. Cell viability was evaluated by MTS assay, after incubation with sEVs derived from patients with LR and patients with IHR before (T0) and after (T1) 8 weeks of VLCKD. Negative controls were untreated cells (CTRL). (** *p* < 0.001 IHR vs. CTRL).

To evaluate the effect of isolated sEVs on the proliferation of HEPA-RG cells, the cells were treated with sEVs derived from patients at T0 and T1 for 96 h. Cell viability was subsequently measured using MTS assays. As illustrated in Figure 7, no significant changes in cell viability were detected in cells treated with sEVs from LR patients. In contrast, a significant decrease in cell viability (*p* < 0.005) was observed in HEPA-RG cells treated with sEVs from IHR patients, compared to the control (CTRL), at both T0 and T1 (Figure 7).

## 4. Discussion

Obesity is a global health concern, and the VLCKD has emerged as a promising option for individuals struggling to achieve weight loss with traditional balanced diets [25,26]. Recent studies have examined the role of EVs in metabolic diseases, particularly their function in transmitting signals to cells involved in hepatic fibrosis progression in obese patients [21]. The use of FibroScan has allowed us to perform an initial stratification of patients based on the degree of fibrosis, particularly as reported in the European Association for the Study of the Liver (EASL) guidelines, patients with a FIB E index < 8, are defined as LR patients, while those with a FIB E index ≥ 8 are defined as IHR for chronic liver diseases [27]. Following this instrumental analysis, 37 patients were categorized as LR, while 23 were classified as IHR. The role of some fatty acids present on the membranes of sEVs in hepatic cell proliferation, cell death and pro-fibrotic increase has been extensively demonstrated using the lipidomic assay [15]. This study confirmed an imbalance between oleic and palmitic acid in both patient groups at baseline (T0) before the VLCKD. After 8 weeks of VLCKD, treatment with T1 sEVs from LR patients restored the balance, while T1 sEVs from IHR patients did not, demonstrating a persistent low viability of the cells treated with IHR-derived sEVs at T0 and T1. Herein, transcriptomic analysis of HEPA-RG cells treated with sEVs revealed that key pathways, including PIK3CB, HDAC10, and MMP19, were significantly influenced. PIK3CB is regulated by both palmitic acid and oleic acid studies indicate that palmitic acid has a detrimental effect on the expression of genes related to the insulin signaling pathway, including PIK3CB, while oleic acid shows more beneficial effects, exerting a reverse control of the negative effect of palmitic acid on insulin resistance and inflammation, thus positively affecting pathways related to PIK3CB signaling, including AKT1 [28,29]. The PI3K/AKT pathway is crucial for cellular growth, survival, and metabolism. Increased PIK3CB and AKT1 activity can reduce HDAC levels, including HDAC3, HDAC6, and HDAC10, through AKT1 phosphorylation, and this reduction has been associated with cellular stress and hepatocyte damage [30,31,32]. Specifically, AKT phosphorylates and inhibits HDAC3 and HDAC6, impacting microtubule acetylation and processes like growth and survival [33,34]. In particular, HDAC6 is pivotal in regulating autophagy, a mechanism vital for cellular waste clearance and stress management. HDAC inhibition disrupts histone deacetylation, increasing inflammatory gene expression and cytokine production, thereby exacerbating inflammation and autophagy deregulation [34]. The findings reported in the literature are strongly supported by our experiments, where the influence of the PI3K/AKT pathway on the modulation of HDACs gene expression is clear. Specifically, an increase in AKT1 phosphorylation is observed in cells treated with sEVs derived from IHR patients at T1. In contrast, in cells treated with sEVs from LR patients, who, despite obesity, do not exhibit a risk of fibrotic degeneration (as evidenced by a FIB E score of less than 8 kPa), and the activity of these molecules remains constant. This underscores the necessity for more stringent monitoring of these patients, even when undergoing a VLCKD.

Metalloproteinases, particularly MMP2, MMP9, and MMP19, regulate the extracellular matrix, which is crucial in multifactorial conditions like obesity and related diseases [35,36,37]. Their reduction in the liver is linked to increased autophagy as a compensatory mechanism to maintain cellular homeostasis and manage stress [38]. Excessive or dysregulated autophagy can trigger autophagic cell death by degrading essential cellular components, reducing cell viability. Its impact depends on the cellular context, degree of activation, and specific stress conditions [39]. In this context, the findings reported in the references are aligned with the results of our experiments. Specifically, while the sEVs from LR patients do not lead to any changes in the expression of the MMPs studied, the sEVs from IHR patients modulate gene expression, which also affects protein expression, suggesting an influence on cellular viability. Indeed, cellular viability is already reduced in cells treated with sEVs from IHR at T0, and this reduction is not restored in cells treated with IHR sEVs at T1, whereas no significantly changes are observed in cells treated with LR sEVs.

Additionally, our experiments revealed a decrease in α-SMA gene expression in cells treated with sEVs from LR patients following VLCKD, indicating a reduction in cellular stress. The same effect was not observed in cells treated with sEVs from IHR patients, in which there was, on the contrary, a marked increase in both α-SMA and Vimentin expression. This demonstrates that cells treated with sEVs from IHR patients undergoing VLCKD continue to express a fibrotic response even after the diet. Moreover, the increased expression levels and delayed response were particularly evident in patients with a FIB E score greater than 8 kPa.

## 5. Conclusions

Previous research has extensively demonstrated that sEVs are crucial mediators of intercellular communication, a role further supported by the findings presented in this study. This is a pilot study in which we have demonstrated the fundamental role of sEVs in early modulation of certain proteins related to inflammation, remodelling, and hepatic cell stress, especially in the cells treated with sEVs from IHR patients before and after the VLCKD. The evidence reported in this study highlights the attention clinicians must pay when evaluating patients before subjecting them to a VLCKD-based dietary regimen. Specifically, the benefits of a VLCKD regimen are well-documented and consistently observed in patients with a low fibrotic risk, in whom we observe a reduction in the expression of fibrosis-related genes and proteins, as well as a modulation of genes linked to regeneration and inflammation like HDACs. In IHR patients, although the VLCKD clearly yielded benefits in terms of weight loss and FIB E reduction, the biochemical and molecular patterns related to inflammation, matrix modulation, and stress fibres appeared to be less responsive to the diet. We are aware of some limitations present in this study, such as certain methodological constraints that are currently in place. It is well known that, although analyses on sEVs are advanced techniques, their standardization and application in clinical practice are still limited. This could hinder the translational interpretation of the results. Furthermore, the study focuses primarily on specific molecular and protein biomarkers (sEVs and related gene/protein modulations), which are useful for understanding biological mechanisms, while overlooking other important aspects, such as the gut microbiota and other systemic inflammatory markers. In light of these encouraging results, and considering the limitation of our study, future opportunities arise for a more in-depth exploration of news molecular mechanisms. These studies could leverage both animal models and an expanded patient cohort, achieved not only through monocentric enrolment but also by broadening the involvement of additional institutions. Future research will focus on tissue inflammatory processes and modifications in metabolic cascades before and after VLCKD, including the microbiota modification, aiming to identify biomarkers that can complement instrumental analyses such as Fibro Scan. This approach will help refine diagnosis, targeted dietary therapy, and clinical follow-up.

## Figures and Tables

**Table 1:** Patient’s characteristics as mean and standard deviation (M ± SD) for continuos variables, and as frequency and percentage (%) for categorical subdivision of all patients into Low Risk (LR) and Intermediate High Risk (IHR) of developing chronic liver disease based on fibrotic score. BMI = body mass index (kg/m^2^); FIB E = fibroscan elastography (kPa); AST = Aspartate Amino transferase (U/L); ALT = Alanine Amino transferase (U/L); γ-GT = Gamma-Glutamyl Transferase (U/L); LDL = Low-Density Lipoprotein (mg/dL); HDL = High-Density Lipoprotein (mg%).

	LR		*p*-VALUE	IHR	*p*-VALUE	
	T0	T1		T0	T1	
GENDER (%)						
M	16 (43.2)	---	---	11(47.8)	---	---
F	21(56.8)	---	---	12 (52.2)	---	---
WEIGHT (kg)	98.1 ± 13.3	89.4 ± 12.3	<0.0001	134.1 ± 25.2	123.1 ± 24.3	<0.0001
BMI (kg/m^2^)	35.9 ± 3.8	32.8 ± 3.6	<0.0001	46.6 ± 6.4	42.0 ± 6.6	<0.0001
FIB E (kPa)	5.1 ± 1.1	4.9 ± 5.3	0.8178	12.1 ± 3.1	7.0 ± 2.8	<0.0001
AST (U/L)	19.9 ± 7.8	20.1 ± 6.2	0.8448	21.3 ± 16.3	28.2 ± 14.2	0.3567
ALT (U/L)	26.2 ± 19.5	24.3 ± 17.9	0.2958	51.5 ± 36.4	46.6 ± 41.4	0.5208
γ-GT (U/L)	24.1 ± 17.9	17.5 ± 9.3	0.0031	37.7 ± 22.2	21.9 ± 12.1	0.0001
FASTING BLOOD GLUCOSE (U/L)	92.3 ± 8.8	86.6 ± 13.8	0.0054	96.0 ± 14.1	88.9 ± 7.6	0.0296
TRIGLYCERIDES (mg/dL)	108.2 ± 57.7	107.9 ± 93.9	0.9833	144.1 ± 93.9	100.9 ± 41.4	0.0234
TOTAL CHOLESTEROL (mg/dL)	182.6 ± 48.7	162.6 ± 47.4	0.0030	182.6 ± 29.9	173.9 ± 124.6	0.7375
LDL (mg/dL)	123.1 ± 36.5	109.0 ± 34.1	0.0094	121.7 ± 24.1	91.3 ± 23.9	<0.0001
HDL (mg%)	51.8 ± 11.6	45.4 ± 10.0	<0.0001	46.8 ± 14.9	42.08 ± 11.4	0.0010

## Data Availability

Sequencing data is available under accession number GSE279581 at the Gene Expression Omnibus (http://www.ncbi.nlm.nih.gov/geo/).

## Supplementary Materials

The following supporting information can be downloaded at https://www.mdpi.com/article/10.3390/nu16234189/s1, Table S1: Membrane composition in HEPA-RG; Table S2: Genes differentially expressed at T1 compared to T0 in HEPA-RG treated with sEVs from patients with LR; Table S3: Genes differentially expressed at T1 compared to T0 in HEPA-RG treated with sEVs from patients with IHR.
