# Supplementary material for: An 8-Week Very Low-Calorie Ketogenic Diet (VLCKD) Alters the Landscape of Obese-Derived Small Extracellular Vesicles (sEVs), Redefining Hepatic Cell Phenotypes
Source: Nutrients. 2024 Dec 4;16(23):4189. doi: 10.3390/nu16234189 (PMC11643994; doi:10.3390/nu16234189)
Supplement: Supplementary file 1 [file nutrients-16-04189-s001.zip › nutrients-3324795-supplementary.pdf]

An 8-Week Very Low-Calorie Ketogenic Diet (VLCKD) alters the landscape of obese derived small Extracellular Vesicles (sEVs), redefining hepatic cell phenotypes .

Francesco Balestra[1†], Maria De Luca[1†], Giorgia Panzetta[1], Nicoletta Depalo [2,3], Federica Rizzi [2,3],Rita Mastrogiacomo[4], Sergio Coletta [5], Grazia Serino[1], Emanuele Piccinno[1], Dolores Stabile[5], Pasqua Letizia Pesole[5], Valentina De Nunzio[6], Giuliano Pinto[6], Nicole Cerabino[7], Martina Di Chito[7], Maria Notarnicola[6], Endrit Shahini[8], Giovanni De Pergola[7†] and Maria Principia Scavo[1†*]

1. Laboratory of Molecular Medicine, National Institute of Gastroenterology IRCCS "S. de Bellis", , Via Turi 27, Castellana Grotte, 70013 Bari, Italy; francesco.balestra@irccsdebellis.it (F.B); maria.deluca@irccsdebellis.it (M.D.L); giorgia.panzetta@irccsdebellis.it (G.P.); maria.scavo@irccsdebellis.it (M.P.S.); grazia.serino@irccsdebellis.it (G.S.); emanuele.piccinno@irccsdebellis.it (E.P.)
2. Institute for Chemical-Physical Processes, Italian National Research Council (IPCF)-CNR SS Bari, Via Orabona 4, 70125 Bari, Italy; n.depalo@ba.ipcf.cnr.it (N.D.); f.rizzi@ba.ipcf.cnr.it (F.R.);
3. National Interuniversity Consortium of Materials Science and Technology (INSTM), Bari Research Unit, Via Orabona 4, Bari, 70126, Italy
4. University of Bari, department of Chemistry, , Via Orabona 4, 70125 Bari, Italy: rita.mastrogiacomo@uniba.it(R.M.)
5. Core Facility Biobank, National Institute of Gastroenterology "S. de Bellis", IRCCS Research Hospital, Via Turi 27, Castellana Grotte, 70013 Bari, Italy; sergio.coletta@irccsdebellis.it (S.C.); dolores.stabile@irccsdebellis.it (D.S.); letizia.pesole@irccsdebellis.it (P.L.P.)
6. Laboratory of Nutritional Biochemistry, National Institute of Gastroenterology, "S. de Bellis", Via Turi 27, Castellana Grotte, 70013 Bari, Italy. valentina.denunzio@irccsdebellis.it (V.D.N.); giuliano.pinto@irccsdebellis.it (Gi.Pi.); maria.notarnicola@irccsdebellis.it (M.N.)
7. Center of Nutrition for the Research and the Care of Obesity and Metabolic Diseases, National Institute of Gastroenterology IRCCS "Saverio de Bellis", Via Turi 27, Castellana Grotte, 70013 BA, Italy; nicole.cerabino@irccsdebellis.it(N.C.); martina.dichito@irccsdebellis.it(M.D.C); giovanni.depergola@irccsdebellis.it(G.DP.)
8. Gastroenterology Unit, National Institute of Gastroenterology IRCCS "S. de Bellis", Via Turi 27, Castellana Grotte, 70013 Bari, Italy; endrit.shahini@irccsdebellis.it (E.S.)

†: These authors contributed equally to this work
* Correspondence: maria.scavo@irccsdebellis.it; Tel.: +39-0804994691

Citation: To be added by editorial staff during production.

Academic Editor: Firstname Lastname

Received: date
Revised: date
Accepted: date
Published: date

Copyright: © 2024 by the authors. Submitted for possible open access publication under the terms and conditions of the Creative Commons Attribution (CC BY) license (https://creativecommons.org/licenses/by/4.0/).

Table S1 Membrane composition in HEPA-RG. The FA composition of HEPA-RG cell membranes was analyzed before (T0) and after (T1) an 8-week period of VLCKD therapy, using sEVs derived from LR and IHR patients. After exposure to LR-derived sEVs, there was an improvement in the FA profile of the cells at T1. However, no significant changes were observed in the FA composition of HEPA-RG cells treated with IHR-derived sEVs at either T0 or T1.

	LR %			IHR %		
	**T0**	**T1**	**p-Value**	**T0**	**T1**	**p-Value**
C4:0 Butyric acid	0.00	0.00		0.00	0.00	
C6:0 Caproic acid	0.00	0.00		0.00	0.00	
C8:0 Caprylic acid	0.04±0.04	0.03±0.02	0.8167	0.06±0.01	0.05±0.01	0.0750
C10:0 Capric acid	1.33±0.14	1.39±0.34	0.7359	1.78±0.19	1.54±0.51	0.5805
C11:0 Undecanoic acid	0.00	0.00		0.00	0.00	
C12:0 Lauric acid	0.19±0.04	0.16±0.03	0.1488	0.39±0.27	0.21±0.02	0.3348
C13:0 Tridecanoic acid	0.07±0.02	0.04±0.02	0.0753	0.09±0.06	0.06±0.01	0.4989
C14:0 Myristic acid	0.64±0.04	0.57±0.05	0.0225	0.92±0.37	0.70±0.12	0.2840
C14:1n5 Myristoleic acid	0.15±0.03	0.13±0.03	0.3080	0.13±0.08	0.16±0.05	0.6067
C15:0 Pentadecanoic acid	0.10±0.01	0.09±0.02	0.3088	0.15±0.06	0.13±0.05	0.6602
C15:1n5 cis-10-Pentadecanoic acid	0.97±0.22	0.81±0.12	0.2489	0.98±0.09	0.95±0.33	0.8771
C16:0 Palmitic acid	17.25±0.76	16.04±0.90	0.0086	17.77±0.90	17.21±3.06	0.7359
C16:1 trans-Palmitoleic acid	0.10±0.04	0.08±0.03	0.4485	0.11±0.02	0.12±0.07	0.9171
C16:1n7 Palmitoleic acid	0.39±0.06	0.36±0.03	0.3586	0.50±0.09	0.44±0.10	0.5434
C16:1n9 cis-9-Palmitoleic acid	1.85±0.18	1.65±0.14	0.0044	2.21±0.12	2.17±0.28	0.8351
C17:0 Margaric acid	0.08±0.01	0.06±0.01	0.0153	0.11±0.07	0.06±0.02	0.4004
C17:1n7 cis-10-Eptadecanoic acid	0.06±0.01	0.05±0.00	0.0993	0.08±0.02	0.06±0.01	0.2159
C18:0 Stearic acid	13.38±0.67	12.84±0.60	0.1543	13.77±0.88	13.34±2.53	0.7364
C18:1 trans Elaidic acid	0.37±0.12	0.43±0.10	0.2700	0.54±0.01	0.46±0.14	0.4119
C18:1n9 Oleic acid	44.23±2.13	49.08±2.49	0.0096	43.65±3.94	45.62±9.85	0.6974

C18:1n7 Vaccenic acid	4.77±0.41	4.16±0.39	0.0006	5.31±0.63	4.73±0.04	0.4126
C18:2 trans Linoelaidic acid	0.19±0.02	0.16±0.02	0.0153	0.27±0.06	0.22±0.03	0.1328
C18:2n6 Linoleic acid (LA)	5.13±0.47	4.37±0.46	0.0061	2.87±0.32	3.64±0.52	0.0261
C18:3n6 γ-linolenic acid (GLA)	0.12±0.01	0.10±0.01	0.0085	0.09±0.04	0.10±0.04	0.7364
C18: 3n3 α-linolenic acid (ALA)	0.01±0.02	0.01±0.02		0.06±0.01	0.03±0.03	0.2743
C20:0 Arachidic acid	0.58±0.05	0.57±0.05	0.2155	0.71±0.06	0.63±0.27	0.6236
C20:1n9 cis-11-Eicosenoic acid	0.59±0.09	0.57±0.07	0.6261	0.56±0.01	0.60±0.14	0.6272
C21:0 Eneicosanoic acid	0.27±0.04	0.21±0.03	0.0396	0.16±0.03	0.17±0.06	0.8893
C20:2n6 cis-11,14-Eicosadienoic acid	0.44±0.05	0.40±0.04	0.1577	0.52±0.06	0.48±0.07	0.1985
C20:3n6 cis-8,11,14-Eicosatrienoic acid (DGLA)	0.53±0.03	0.42±0.04	0.0133	0.42±0.02	0.42±0.13	0.9911
C20:4n6 Arachidonic acid (AA)	3.78±0.19	3.11±0.19	0.0047	3.16±0.39	3.16±0.87	0.9938
C20:3n3 cis-8,11,17-Eicosatrienoic acid (ETE)	0.00	0.00		0.00	0.00	
C22:0 Behenic acid	0.11±0.01	0.10±0.02	0.6014	0.12±0.01	0.17±0.07	0.2847
C22:1n9 Erucic acid	0.08±0.02	0.08±0.02	0.7322	0.14±0.11	0.18±0.17	0.8140
C20:5n3 Eicosapentaenoic acid (EPA)	0.21±0.02	0.20±0.02	0.1919	0.22±0.02	0.21±0.06	0.8040
C23:0 Tricosanoic acid	0.03±0.03	0.03±0.02	0.2190	0.12±0.09	0.07±0.01	0.3574
C22:2n6 cis-13,16-ocosadienoic acid	0.00	0.00		0.00	0.00	
C24:0 Lignoceric acid	0.08±0.02	0.07±0.01	0.2382	0.12±0.01	0.11±0.03	0.7696
C24:1n9 Nervonic acid	0.24±0.04	0.20±0.03	0.0227	0.28±0.02	0.29±0.05	0.9580
C22:5n3 Docosaepentaenoic acid (DPA)	0.64±0.02	0.58±0.07	0.1202	0.67±0006	0.65±0.14	0.8485
C22:6n3 Docosaehenanoic acid (DHA)	0.98±0.08	0.84±0.06	0.0284	0.96±0.09	0.87±0.20	0.3116

Table S2 Genes differentially expressed in T1 compared to T0 in HEPA-RG treated with sEVs from LR patients.

ID	Fold Change	P-value
LOC389332	1.58	0.0003
METAP1D	-1.56	0.0062
DYSF	1.62	0.0068
FMO5	-1.55	0.0078
POU2F2	-1.61	0.0090
CMC1	1.64	0.0110
DNM3	-1.51	0.0152
PKDCC	-1.61	0.0200
SHOX2	1.66	0.0211
RYR1	1.53	0.0219
IL21R	1.67	0.0225
FKBP1AP1	-1.64	0.0226
ASIC3	1.54	0.0258
AP1AR	1.51	0.0293
ARHGAP26	-1.59	0.0377
FAM65A	1.51	0.0414
MGAT5B	-1.60	0.0417
GHRLOS2	-1.64	0.0418
RXFP4	-1.54	0.0451

Table S3 Genes differentially expressed in T1 compared to T0 in HEPA-RG treated with sEVs from IHR patients.

ID	Fold Change	P-value
MEGF6	-1.73	0.000
FSTL4	-1.60	0.000
PPP3CC	1.56	0.001
LDOC1L	-1.74	0.001
LOC100289019	-1.55	0.002
TMEM237	1.60	0.002
DPY30	2.18	0.002
ARL14EPL	1.52	0.003
HOTAIRM1	1.60	0.003
ADCK5	-1.78	0.003
SFN	-1.66	0.003
MORF4L1	1.50	0.003
C9orf37	-1.65	0.003
SNORA5C	-1.77	0.004
EML5	-1.65	0.004

HSD3BP4	-2.28	0.004
OSCAR	-1.61	0.005
DNAH1	-1.63	0.005
SEMA5A	-1.70	0.006
PRTFDC1	1.51	0.006
TWISTNB	1.66	0.006
TAPT1	1.57	0.007
PIK3CB	1.60	0.007
LOC100288069	-1.58	0.007
PRSS27	-1.59	0.007
ANXA2P1	-1.58	0.007
PDE1B	-1.61	0.007
RILPL1	-1.68	0.008
NOXA1	-1.65	0.008
PITPNM3	-1.53	0.008
SPATA7	1.58	0.008
FAM160B2	-1.60	0.008
PDE8B	1.60	0.008
THAP8	-1.67	0.008
PLK3	-1.59	0.009
ZNF513	-1.55	0.009
GGT7	-1.63	0.009
ANTXR1	-1.52	0.009
KRTAP3-1	-1.59	0.009
USP17L6P	-1.54	0.009
LOC100128361	-1.78	0.009
TSNARE1	-1.61	0.009
KCNQ4	-1.84	0.009
CDRT15P1	-1.70	0.010
APCDD1L-AS1	1.57	0.010
FAM27A	-1.54	0.010
LYRM9	1.51	0.010
HDAC10	-1.67	0.010
UNKL	-1.51	0.011
HSD3B7	-1.84	0.011
NUDT8	-1.63	0.011
TTC29	-1.62	0.012
TAS2R39	-1.50	0.012
C2orf81	-1.84	0.012
PDDC1	-1.65	0.012
MAB21L2	-1.69	0.013
ZNF154	-1.55	0.013
ZNF25	1.73	0.013
LINC00649	-1.57	0.013
SERPINB5	1.57	0.013
SOCS3	-1.86	0.014

MON1B	-1.59	0.014
ZNF594	-1.55	0.014
MEF2A	1.71	0.014
OSBPL7	-1.64	0.015
C15orf62	-1.65	0.015
RCN3	-1.54	0.015
EOMES	-1.55	0.015
FAM86B1	-1.54	0.015
AGSK1	-1.72	0.017
GOLGA2P5	-1.96	0.017
ADAMTSL2	-1.51	0.017
LOC401321	-1.68	0.017
GLTP	-1.54	0.018
SLC35G3	-3.05	0.018
CACNG6	-1.59	0.018
HIST1H4I	-1.59	0.019
MYPOP	-1.61	0.019
PPFIA4	-1.55	0.019
CNTF	-1.63	0.019
COA6	1.53	0.019
SNORA70G	-2.42	0.020
CD74	-1.59	0.020
C17orf103	-1.59	0.020
UPK3BL	-1.53	0.021
PAM16	-1.61	0.021
TMEM62	1.60	0.021
FOXK1	-1.54	0.021
OR51I1	-1.72	0.021
LOC100131089	1.63	0.022
CHCHD1	2.04	0.022
TRIM9	-1.72	0.022
SLC27A5	-1.71	0.023
SH3RF3-AS1	-1.63	0.023
F10	-1.58	0.023
ANO8	-1.54	0.023
LRRC8A	-1.95	0.024
TCTEX1D2	1.51	0.024
CHD2	-1.54	0.025
MMADHC	1.52	0.025
ANO9	-1.50	0.025
LOC645166	1.66	0.025
CHTF18	-1.55	0.025
TOB2	-1.51	0.025
SNORA14A	-1.90	0.025
C1QTNF1	-1.55	0.026
MAPRE3	1.80	0.026

GPATCH2	-1.52	0.026
NBPF4	1.70	0.026
RASAL1	-1.50	0.026
RPL13AP3	-1.53	0.026
VPS37D	-1.62	0.027
SLC43A2	-1.63	0.027
SLC6A12	-1.58	0.027
ASF1A	1.55	0.028
PRSS16	1.54	0.028
PPIL3	1.71	0.028
ATP9B	-1.67	0.029
SEC22A	1.50	0.029
APLN	-1.52	0.029
LOC150776	-1.56	0.030
DKFZP434H168	-1.78	0.030
HSF4	-1.97	0.031
GPR146	-2.48	0.032
COL4A4	-1.67	0.032
HSPD1	1.53	0.033
HSP90AB2P	-1.65	0.033
COX6B2	-1.64	0.033
CTSW	-1.65	0.034
GPER	-1.78	0.034
LOC100506888	-2.75	0.034
SRCIN1	-1.71	0.035
AMPD2	-1.52	0.035
PDLIM2	-1.56	0.035
MRPS35	1.73	0.036
BAIAP3	-1.67	0.036
HSPA14	1.63	0.037
POLE2	1.55	0.037
NTN4	-1.55	0.037
ADAT3	-1.70	0.037
TMOD1	-1.51	0.038
C9orf53	-1.89	0.038
RNF5	-1.52	0.039
PIEZO1	-1.51	0.039
CEP70	1.52	0.039
TOB2P1	-1.68	0.040
PDP2	-1.79	0.041
DLEU1	1.81	0.042
HSD17B1	-1.73	0.042
MMP19	-1.53	0.043
WDR85	-2.16	0.043
VANGL2	-1.52	0.044
IGSF9B	-2.00	0.045

OXER1	-1.77	0.045
ZNF444	-1.69	0.045
LOC100126784	-1.69	0.046
N4BP3	1.67	0.048
HOXA7	-1.66	0.048
TYRO3P	-1.97	0.048
NRP2	-1.55	0.049
PPOX	-1.64	0.049
MIRLET7BHG	-1.75	0.049
EGFL8	-2.34	0.049
RPL13AP6	-1.81	0.050
